# Microbial community responses to soil tillage and crop rotation in a corn/soybean agroecosystem

**DOI:** 10.1002/ece3.2553

**Published:** 2016-10-14

**Authors:** Chris R. Smith, Peter L. Blair, Charlie Boyd, Brianne Cody, Alexander Hazel, Ashley Hedrick, Hitesh Kathuria, Parul Khurana, Brent Kramer, Kristin Muterspaw, Charles Peck, Emily Sells, Jessica Skinner, Cara Tegeler, Zoe Wolfe

**Affiliations:** ^1^ Department of BiologyEarlham CollegeRichmondINUSA; ^2^ School of Natural Science and MathematicsIndiana University EastRichmondINUSA; ^3^ Department of Computer ScienceEarlham CollegeRichmondINUSA; ^4^ Present address: Department of EntomologyUniversity of IllinoisUrbana ChampaignILUSA

**Keywords:** 16S rRNA, crop rotation, MG‐RAST, microbial ecology, mothur, shotgun sequencing, tillage

## Abstract

The acreage planted in corn and soybean crops is vast, and these crops contribute substantially to the world economy. The agricultural practices employed for farming these crops have major effects on ecosystem health at a worldwide scale. The microbial communities living in agricultural soils significantly contribute to nutrient uptake and cycling and can have both positive and negative impacts on the crops growing with them. In this study, we examined the impact of the crop planted and soil tillage on nutrient levels, microbial communities, and the biochemical pathways present in the soil. We found that farming practice, that is conventional tillage versus no‐till, had a much greater impact on nearly everything measured compared to the crop planted. No‐till fields tended to have higher nutrient levels and distinct microbial communities. Moreover, no‐till fields had more DNA sequences associated with key nitrogen cycle processes, suggesting that the microbial communities were more active in cycling nitrogen. Our results indicate that tilling of agricultural soil may magnify the degree of nutrient waste and runoff by altering nutrient cycles through changes to microbial communities. Currently, a minority of acreage is maintained without tillage despite clear benefits to soil nutrient levels, and a decrease in nutrient runoff—both of which have ecosystem‐level effects and both direct and indirect effects on humans and other organisms.

## 1 Introduction

Of the lands dedicated to agriculture in the United States, the cultivation of corn and soybeans accounted for approximately 174 million acres in 2015, an area roughly the size of Texas (National Agriculture Statistics Service, United States Department of Agriculture, 2015). In addition, corn and soybean harvests constituted approximately 1% of the US gross domestic product in 2014 (National Agriculture Statistics Service, United States Department of Agriculture, 2015). How the soil is treated in these agroecosystems, therefore, can have major impacts on global food production, the livelihood of farmers and those dependent on them, and large‐scale effects on ecosystem health and services. Many agricultural practices, such as crop rotation and no‐till methods, are directed at maintaining nutrients within the soil and thus decreasing the need for supplements. Minimizing soil disturbance by not tilling has been shown to decrease soil erosion (and nutrient runoff into water bodies) and the energy needed for agriculture, while not having a major impact on crop yield (DeFelice, Carter, & Mitchell, 2006; Phillips, Blevins, Thomas, Frye, & Phillips, 1980). In contrast, not tilling increases soil compaction (Håkansson, Voorhees, & Riley, 1988) and can indirectly increase the need for herbicides (Gersmehl, 1978), both of which can have negative impacts on crops and the surrounding environment. Furthermore, nutrient loss from tilling is lessened in soils that are less susceptible to erosion (Denton & Wagger, 1992; Wagger & Denton, 1992). Only 20–35% of corn/soybean farms in the United States used no‐till practices in 2009.

Microbial communities can be directly affected by tilling due to habitat modifications (pore space), the loss of connectivity of individuals and species, disruption of physical networks of nutrient passage, and by increased runoff (Young & Ritz, 2000). Soil microbial communities are the drivers of nutrient cycling, including those nutrients that most limit crop growth, nitrogen, and phosphorus. Therefore, in addition to affecting nutrient levels via erosion, soil tillage may have indirect effects on nutrient levels via effects on microbial communities (Mathew et al., 2012). Furthermore, microbial communities can drive patterns of greenhouse gas emission via their roles in nutrient cycling (e.g., nitrous oxide production). Human‐mediated changes to microbial community structure and function are well documented (Andrade, Colozzi‐Filho, & Giller, 2002; Ceja‐Navarro, Rivera et al., 2010; Ceja‐Navarro, Rivera‐Orduna et al., 2010; Garbeva, van Veen, & van Elsas, 2004; Govaerts et al., 2008; Jansa et al., 2003; Souza et al., 2015; Stromberger, Shah, & Westfall, 2007), but little is known about the relationship between community composition and function.

The frequent disturbances associated with tillage may drive patterns of soil diversity by favoring species that are better dispersers or tolerators of disturbance, at the expense of the better competitors (Buckling, Kassen, Bell, & Rainey, 2000; Connell, 1978), thus not just affecting the composition of the communities, but also their diversity. More diverse communities (including both taxonomic and functional diversity) tend to be more stable in the face of environmental variation as well as more resistant to invasion by other species (Eisenhauer et al., 2012; van Elsas et al., 2012; Tilman et al., 1997). However, community diversity and response to disturbance are highly nuanced (Allison & Martiny, 2008) and vary with the type and severity of disturbance (Hobbs & Huenneke, 1992), the timescale studied (Wertz et al., 2007), and on the starting identity of the initial community (Chase, 2003).

In this study, we used both 16S rRNA amplicon sequencing and shotgun sequencing to evaluate how conventional tilling and crop rotation affect soil bacterial community structure and function. We took advantage of the common practice of annual corn–soybean crop rotation and the pairing of rotated fields (i.e., physically proximate fields of similar size are kept in opposite crops). Using whole fields that were all part of commercial operations, our results are likely generalizable, but this resulted in less control over specific field management (cover crops, herbicide/pesticide use, fertilizer use, etc.). We hypothesized that the soil disturbance associated with conventional tillage would cause greater turnover of bacteria, disrupting community structure and function and also causing a decrease in community diversity and composition. We also hypothesized that nutrient levels would differ consistently between fields that were tilled and not and that such differences would in turn correspond with bacterial community structure and function. As bacterial communities are primarily responsible for the biotic breakdown and cycling of nutrients, we believe that our results bear insights on the interaction between soil nutrients and bacterial communities in agricultural fields, both of which impact human and environmental welfare.

## 2 Methods

### 2.1 Site description and sampling design

Twenty agricultural fields were sampled in early July, 2012 and 2013. The same fields were sampled in each year. A field sample consisted of five 10‐cm‐deep soil cores (2.5 cm diameter) distributed evenly across the field that were then pooled, mixed, sampled for DNA, and sent for chemical analysis. All fields were near Richmond, Indiana (Appendix Table S1). In 2012, half of the sampled fields were planted in corn and the other half in soybeans; each corn field was paired with a soybean field of approximately equal size operated by the same farmer. Half of the fields were operated by farmers that self‐identified as using conventional tilling practices, while the other half by farmers identifying as using no‐till practices. The strain of crop, as well as the type and amount of fertilizer (which included hog, chicken, and treated human waste, as well as formulated liquid fertilizer) and pesticides, varied greatly among farmers and fields. In 2013, the same fields were sampled, although the crop was rotated from corn to soybean, or vice versa; however, two previously sampled fields were not resampled as they had been rotated to wheat, and one was resampled although it was replanted in consecutive corn.

Soil (300–500 g) was sent for chemical analyses at A and L Great Lakes Laboratory (Muncie, Indiana). Analyses included total organic carbon, available phosphorus, exchangeable potassium, magnesium, calcium, and total nitrogen (Dumas method). Percent soil moisture was calculated by drying soil in an oven at 55°C for at least 48 hr and dividing the difference in wet and dry soil mass by the wet soil mass; each measurement was carried out in duplicate and averaged.

### 2.2 DNA extraction and sequencing

DNA was extracted from the soil using the MO‐BIO Power Soil^®^ kit using 0.25 g of soil. Extracted DNA was quantified using the Quant‐iT™ Picogreen dsDNA kit (Invitrogen) using the protocol described by Blotta, Prestinaci, Mirante, and Cantafora (2005), and DNA was sent to the Genome Sequencing Facility at the Greehey Children's Cancer Research Institute for shotgun sequencing. Shotgun sequencing libraries from 2012 and 2013 were prepared and sequenced in one lane on an Illumina HiSeq (2 × 100PE). Detailed library preparation methods, from the sequencing facility, are described in the Appendix (Detailed Shotgun Library Preparation Methods). Barcoded 16S rRNA amplicon libraries of region V4 were prepared from extracted DNA from both 2012 and 2013 and prepared using the protocols described by Caporaso et al. (2012). Primers 515F and 806R were used in all 16S rRNA amplifications, with 806 carrying a field‐specific barcode (see Appendix Table S2 for individual barcodes used). PCR was carried out in 25‐μl reaction tubes with 0.2 μl Accuprime Taq (Invitrogen), 1X Accuprime buffer I, 0.2 μmol/L of each primer (515F and 806R), and 10 ng of DNA per PCR. PCR products were cleaned using Agencourt AMPure XP paramagnetic beads (Beckman Coulter, Inc.), quantified using Quant‐iT™ Picogreen (Invitrogen), as above, combined at equimolar ratios, and sequenced in two 2 × 150 PE MiSeq runs with 300 cycles (samples from 2012 and 2013 were sequenced in separate runs) at the Center for Genomics and Bioinformatics at Indiana University, Bloomington.

### 2.3 16s rRNA copy number

The number of 16S rRNA copies in each sample was quantified using qPCR relative to *Escherichia coli* plate counts (Irwin et al., 2014). The number of colony‐forming units (CFU) of *E. coli* strain K‐12 (Carolina Biological Supply) was quantified using serial dilutions (plates were duplicated and the average used in calculations); there were 9.1 × 10^8^ CFU/ml.DNA from the same *E. coli* was then isolated using a MO‐BIO UltraClean Microbial DNA isolation kit; an average of 2.6 μg of DNA was extracted from duplicate extractions. For simplicity, we assumed 100% efficiency of DNA extraction, and thus, the following calculations err on the side of underestimation of 16S rRNA copy number. *Escherichia coli* strain K‐12 has seven rRNA operons (Ellwood & Nomura, 1982). Thus, there were 6.2 × 10^5^ CFU/ng of DNA or 4.3 × 106 copies of 16S rRNA per ng of DNA in our populations of *E. coli* strain K‐12. To calculate the number of 16S rRNA copies in our samples, we first quantified 16S rRNA copy number relative to a fourfold serial dilution of the above *E. coli* using qPCR—and the above relationship between copy number and ng of DNA for these *E. coli*: reactions were 10 μl using 5 μl of QuantiTect SYBR green real‐time PCR master mix (Qiagen), 0.3 μmol/L of each primer (515F and 806R, as above), and 10 ng of template DNA, and run in duplicate and the average C_t_ used in calculations; reactions were run on an Eppendorf Realplex real‐time PCR machine. The number of copies per ng of DNA was then converted to copies per mg of soil using the quantity (as estimated from a Nanodrop 2000c spectrophotometer) of DNA extracted per mg of wet soil and then corrected for dry soil mass based on soil moisture measurements. The amount of DNA extracted from soil was consistent, slope = 0.98 and *r* ^2^ = .78, from independent extractions of the same soil. It is unknown whether the soils used in this study differed sufficiently to affect extraction efficiency, but such differences could have affected these calculations.

### 2.4 Bioinformatics

Shotgun sequencing reads were processed using the MG‐RAST pipeline (Meyer et al., 2008) using default parameters and filtering out human sequences, see Appendix Table S3 for library details and accession numbers. Field 18 from 2012 produced far less sequence than all other libraries and was excluded from analyses. Functional annotations, level three, using the SEED database at MG‐RAST were downloaded and processed using the BIOM (McMurdie, 2014) and vegan (Oksanen et al., 2013) packages for R (R Core Team, 2014), see below for analysis details. All data were normalized and the quantities reported are the number of annotated reads (for the particular function) per million sequenced reads.

16S rRNA sequences were processed separately for each year using mothur (Schloss et al., 2009) following the standard operating procedure described previously (Kozich, Westcott, Baxter, Highlander, & Schloss, 2013). No “mock” community was used in our analysis and so error rate could not be estimated. Operational taxonomic units (OTUs) used in all downstream analyses were based on 97% sequence identity. Years were processed separately due to computational load. Years were merged based on taxonomy for analyses that required both years together. In order to rarify, data were subsampled (see Appendix Table S3 for library details) to the lowest common sequence count within years.

### 2.5 Statistical analyses

#### 2.5.1 Alpha diversity

Analyses on alpha diversity were performed in R (R Core Team, 2014) using a repeated‐measures analysis of variance (ANOVA) with both tillage type and crop as factors and repeated samples (fields) within tillage type as the repeated measure.

#### 2.5.2 Beta diversity

We compared means (one sided *t*‐test) of between year dissimilarities (both Bray–Curtis and Sorensen) of conventionally tilled and no‐till fields to test for species turnover (from 16S rRNA OTU data) between years. That is, from the distance matrix of all fields, was the between‐year distance for each field different between no‐till and conventionally tilled fields?

Nonmetric multidimensional scaling (NMDS—”metamds” function), permutational ANOVA (“adonis” function), and similarity percentage analysis (SIMPER—”simper” function) used the vegan package (Oksanen et al., 2013) for R. Distance matrices were constructed using Bray–Curtis distances in the function “vegdist”. Indicator species were predicted using the “multipatt” function with 999 permutations in the indicspecies package for R (De Caceres, Jansen, & De Caceres, 2015).

We examined variation at higher taxonomic levels by examining skew between conventionally tilled and no‐till fields. We calculated skew as the difference between abundances across conventionally tilled and no‐till fields. Only phyla with at least ten genera were included in the analysis. A chi‐square goodness‐of‐fit test was used to test for bias where expected values represented no bias (i.e., half of all taxa biased in each direction).

#### 2.5.3 Soil chemistry

**Figure 1:**
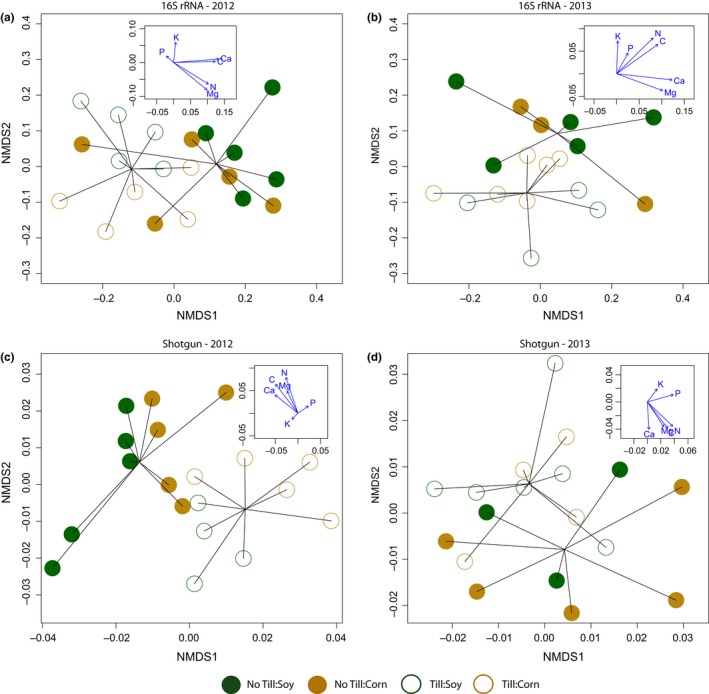
Ordination (NMDS) plots of communities for each year and using 16S rRNA amplicons (a, b) and functional shotgun annotations (c, d). Insets show correlations of axes with soil nutrients. There was a statistically significant difference between tilled and untilled fields in all years and across both data types (16S rRNA and shotgun); crop type was only statistically significant for shotgun/function in 2012 (Appendix Table S8). Soil nutrients tend to increase in the direction of no‐tillage fields in all four panels (Appendix Figure S4)

We hypothesized that conventional tillage would cause an overall decrease in soil nutrients and that this in turn would help explain any observed differences in field bacterial communities. All elements assayed were correlated with NMDS axes and plotted as vectors on the NMDS plots of communities (Figure 1) as an exploratory analysis. Further, the degree of correlation among all pairs of soil nutrients was assessed. Differences between crop and tillage were tested for all of the elements assayed using repeated‐measures ANOVA (crop and tillage as independent variables repeated over years). Additionally, a combined multivariate analysis of variance (MANOVA) was used to examine the effects of crop and tillage type on combined soil nutrients, that is, tillage may have the same effect on all nutrients due to runoff, but this effect may be obscured in individual analyses. As above, these analyses were performed using R (R Core Team, 2014), but multivariate outliers were assessed using the “aq.plot” function of the mvoutlier package (Filzmoser & Gschwandter, 2015).

#### 2.5.4 Nitrogen cycle

We analyzed the functional annotation terms (from MG‐RAST annotated shotgun sequencing, described above) associated with the nitrogen cycle and compared normalized values, annotated reads per million, across field tillage type and crop using repeated‐measures ANOVA (years were repeated measures).

## 3 Results

### 3.1 Alpha diversity

The average field had over 23,000 operational taxonomic units (OTUs) after rarefaction, and over all fields, before rarefaction, there were between 278,626 (2012) and 292,327 (2013); note after rarefaction, the gamma diversity decreased and was more similar between years, 250,039 and 249,811 OTUs in 2012 and 2013, respectively. These numbers decrease dramatically when singletons are excluded—singleton sequences can represent both rare OTUs as well as sequencing and sequence processing errors. The total number of OTUs in each year, excluding singletons, was 70,507 (2012) and 64,422 (2013); the number of sequences in the average field fell to 14,601.

Fields that were conventionally tilled had a greater number of OTUs compared to no‐till fields (*F*_1,32_ = 8.5, *p* < .01), although this result is only marginally significant when singletons are excluded from the analysis (Appendix Tables S4 and S5). However, abundance, richness, and diversity were not significantly different between tillage types and crops for either shotgun‐annotated species or shotgun‐annotated function; see Appendix Tables S4–S7 for detailed information on each of the metrics used.

On average, fields were estimated to have greater than 27 million copies of 16S rRNA per gram of dry soil. While there was no difference with regard to tillage type or crop, there was a significant difference in 16S rRNA copy abundance between years (2012: 36,580,108 copies, 2013: 17,433,760 copies, *t*_36_ = 5.4, *p* < .0001). While samples were taken at the same time of year and time of day in both years, 2012 was a dry year compared to 2013 and samples from each year did differ in soil moisture (2012: 6% water, 2013: 15% water, *t*_36_ = 16.2, *p* < .0001). However, when soil moisture was analyzed separately for 2012 and 2013, both tillage type and crop were statistically significant or marginal (*F*_1,16_ > 3.5, *p* < .08 in all cases; the interaction term was not significant in any case) where soybean fields (relative to corn) and no‐till fields (relative to conventional till) had higher moisture (Appendix Figure S1).

### 3.2 Beta diversity

We hypothesized that the tilling of soil would disturb communities, thus increasing their dissimilarity (higher species turnover). There was no evidence of increased turnover due to soil tillage for either distance metric (*p* > .4).

Nonmetric multidimensional scaling plots (Figure 1) show clear partitioning between conventionally tilled and no‐till fields for both years and for both 16S rRNA taxonomic diversity and MG‐RAST annotated functional diversity (using level 3 functional annotations). There is also a clustering of crops within each till group, but this is less clear. The observed clustering is robust to the inclusion of singleton sequences (Appendix Figure S2 shows the same plots excluding singletons). The statistical significance of multivariate grouping was analyzed using permutational ANOVA for distance matrices (crop and tillage as factors). In both years, there was a significant effect of tillage on 16S rRNA community grouping (*F*_1,16_ = 4.1, *F*_1,14_ = 1.8, *p* < .005 and *p* < .05 for 2012 and 2013, respectively), but not of crop or the interaction between crop and tillage type (Appendix Table S8). Similarly, there was a significant effect of tillage on community grouping by annotated function in both years (*F*_1,15_ = 6.9, *F*_1,14_ = 2.1, *p* < .001, *p* < .05 for 2012 and 2013, respectively); crop type also significantly affected functional grouping, but only in 2012 (*F*_1,15_ = 4.0, *p* < .005; Appendix Table S8).

We used several methods to investigate whether particular taxonomic groups were overrepresented or predictive for either conventionally tilled or no‐till fields, similarity percentage analysis (SIMPER), indicator species analysis, and an analysis on phylum‐level bias.

**Figure 2:**
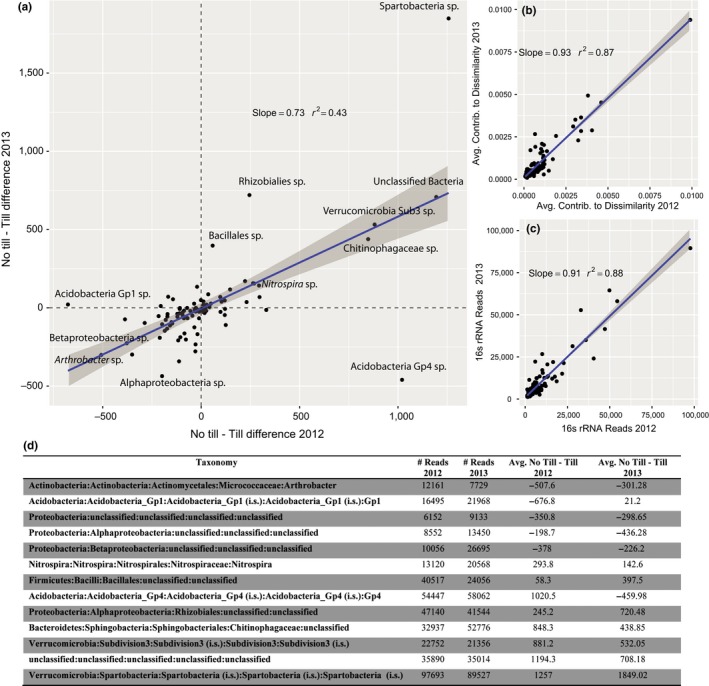
Changes in the most dominant taxa from year to year. In (a), the axes are the difference in abundance for each taxon between no‐tillage and conventionally tilled fields (i.e., taxa with higher abundance in no‐till fields are positive). In (b), taxa are plotted by their average contribution to community dissimilarity (comparing conventionally tilled and no‐till fields, from SIMPER analysis) for each 2012 and 2013. Panel (c) is the relationship between taxon abundance (number of reads, after rarefaction) between years. Panel (d) is a table with the taxonomy of the most influential (and abundant) taxa for differentiating conventionally tilled and no‐till fields. Taxa are ordered by their bias toward no‐till fields. The slopes for the plots (a–c), are all highly statistically significant, *p* < .0001

The SIMPER analysis was performed separately on each year in order to minimize the influence of year‐to‐year variation. Results from each year were then combined by taxonomy and compared to find consistently high “leverage” taxa (Figure 2a). The difference in average OTU abundance between no‐till and conventional till groups was used as a metric for OTU association with tillage (Figure 2a). There was consistency between years in taxon contribution to community dissimilarity (Figure 2b) and taxa were consistent in their normalized abundance between years (Figure 2c). Both the average contribution of OTUs to community dissimilarity and the average abundance of OTUs between years were highly correlated (*t* = 25.09 and 25.55, *df* = 88, *p* < .0001 for both average dissimilarity and abundance, respectively, Figure 2b,c). A type of Spartobacteria and an unclassified bacteria were most highly associated with no‐till fields, while *Arthrobacter* sp. were most associated with tilled fields; Figure 2d has a full listing of the most different taxa.

The indicator species analysis found six OTUs predictive (at *p* < .05) of no‐till fields and 19 predictive of conventional tilled fields; however, the majority of these had very low abundance. These taxa were representative of major soil phyla (Appendix Table S9).

Some phyla showed a very high skew in their abundance across conventionally tilled and no‐till fields. Actinobacteria, Firmicutes, and Planctomycetes had many more than expected biased taxa in no‐till fields (χ^2^ = 39.2, 33.2, and 4.5, *df* = 1, *p* < .0001, .0001, and .05, respectively), while Bacteroidetes and Proteobacteria had more biased taxa than expected in tilled fields (χ^2^ = 5.7 and 35.1, *df* = 1, *p* < .05 and .0001, respectively, Appendix Figure S3).

### 3.3 Soil chemistry

The insets in Figure 1 show the correlation between the NMDS axes and nutrient levels. There is a consistent pattern of increased levels of most nutrients with no‐till fields. Most nutrients were also correlated with each other (13 of the 15 pairs had positive correlations, and nine of these were statistically significant at *p* < .05). There was no statistically significant effect of crop type, tillage, or their interaction in any univariate comparisons (Appendix Figure S4). However, given the degree of correlation among nutrients and their pattern of increase in no‐till fields, it is not surprising that tillage type is significant in a MANOVA using all elements as dependent variables (approximate *F*_6,27_ = 4.7, *p* < .005), but neither crop nor the interaction between crop and tillage type were statistically significant (however, this test was performed with a known departure from multivariate normality, as judged by Mahalanobis distances, and should be interpreted with caution).

### 3.4 Nitrogen cycle

**Figure 3:**
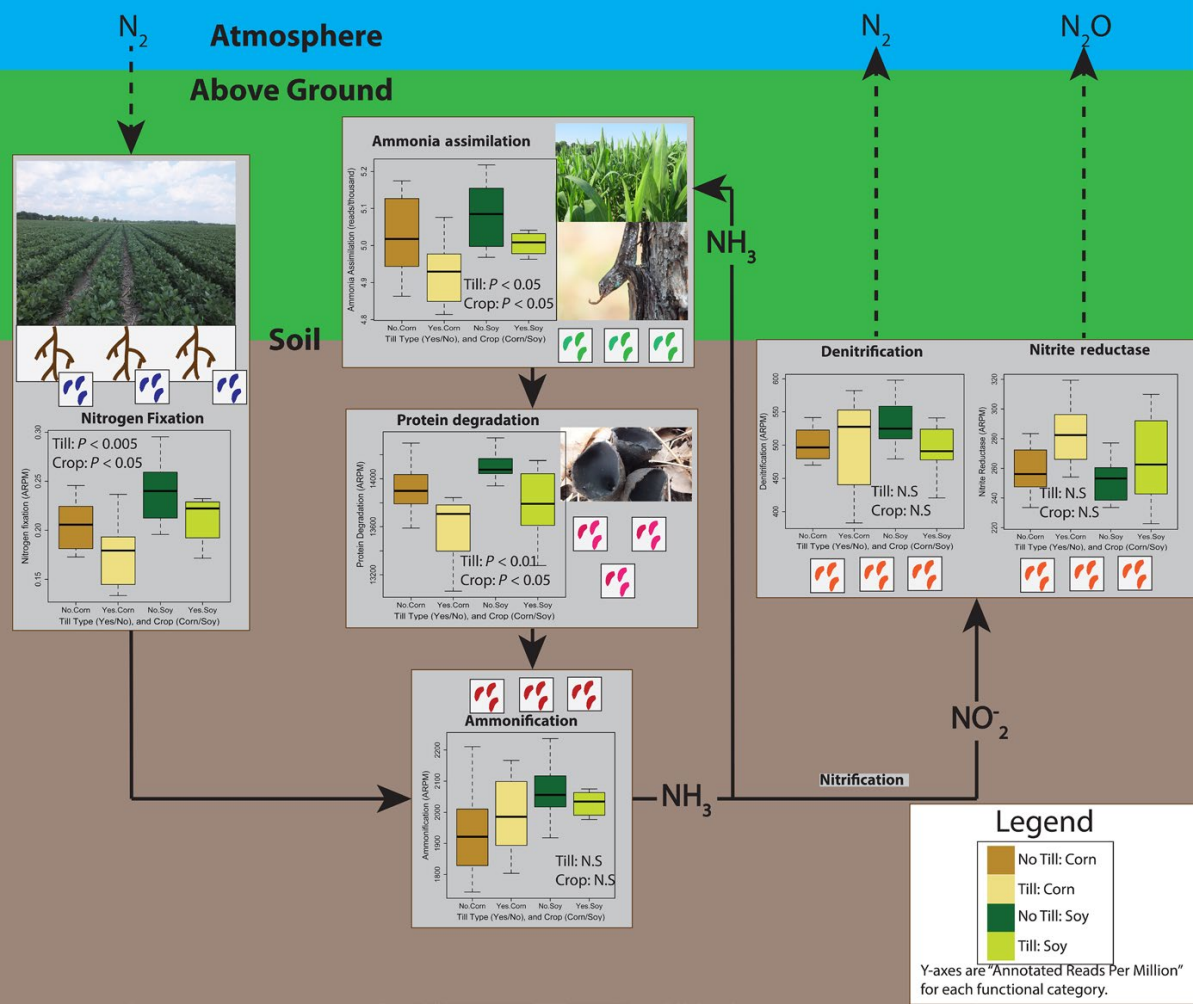
A schematic of changes in normalized annotated reads (annotated reads per million) for different nitrogen cycle processes across the two factors: crop type and tillage regime. The position of each box corresponds to where (atmosphere, above ground, or soil) the process is occurring, and the pictures correspond to what groups of organisms are chiefly responsible (colored dots are used to represent bacteria). *p*‐Values are from repeated‐measures ANOVA; box plots are median (line), interquartiles (box), and range (whiskers)

These results are summarized in Figure 3. Not surprisingly, there was a significant difference between soybean and corn soil bacteria in DNA for nitrogen fixation, with more N‐fixation genes present in soybean fields (*F*_1,31_, *p* < .005). Soybean fields also had more annotated terms for ammonia assimilation and protein degradation (*F*_1,31_ = 5.9 and 7.3, respectively, *p* < .05 for both). Interestingly, no‐till fields also had significantly more reads with ammonia assimilation and protein degradation terms than conventionally tilled fields (*F*_1,31_ = 7.2 and 9.1, *p* < .05 and *p* < .01, respectively). Interaction terms were not significant for any functional categories. Neither crop nor tillage type were significant factors for other N‐cycle terms. One interesting trend, though not statistically significant, is the increase in dissimilatory nitrite reductase reads in tilled compared to no‐till fields; this is the only annotated nitrogen cycle term with the reverse pattern compared to the others, with higher median counts in no‐till fields (Figure 2). Overall, this analysis of nitrogen cycle functional annotation terms suggests a more active nitrogen cycling bacterial community in no‐till fields.

## 4 Discussion

Our results demonstrate clear differences in bacterial community composition, function, and soil nutrient profiles between commercially farmed fields that were either conventionally tilled or where farmers practiced no‐till soil management. The effect of soil tilling was much more evident in all analyses than the crop that was planted, consistent with previous studies (Buckley & Schmidt, 2001, 2003; Jangid et al., 2011), and this effect was consistent across independent methods of assaying for diversity (16S rRNA and shotgun DNA sequencing). The correlation between nutrient levels and bacterial processes (as inferred from DNA annotations) provides intriguing evidence that tilling soil affects both abiotic and biotic components of ecosystem‐level processes. The consistency and magnitude of the observed effect of tillage is surprising given the many variables among farms, including the types of fertilizer (including hog manure, chicken manure, treated human waste, and liquid formulae), pesticide use, and field size.

The deep sequencing of agricultural soil represented in this study agrees with the previous estimates of both bacterial diversity and abundance in soils. We detected many millions of 16S rRNA amplicons per gram of soil, and nearly 300,000 unique OTUs (at the 3% cutoff, singletons included) across our relatively small sampling area in eastern Indiana. Our results for OTU number, after the removal of singletons, are similar to other reported results using similar methods (Fierer et al., 2012).

We found that conventionally tilled fields had more OTUs than no‐till fields. This result is contrary to our hypothesis, but previous studies show highly variable results (Andrade et al., 2002). Furthermore, the different field types had very little difference in richness or diversity (Appendix Tables S4–S7), suggesting that crop type and tillage have negligible effects on the number and diversity of species—but instead affect species composition.

Although we show clear differences in community composition between conventionally tilled and no‐till fields, the differences appear mostly driven by changes in the abundance of species shared by both field types (generalists) rather than specialists that were unique to one of the field types. For this reason, the taxa most driving differences in community composition are among the most abundant while indicator species of tillage are relatively rare (Table S7). The most abundant taxa were consistent between both years, regardless of the crop being grown (Figure 2c). The affects of tillage appear to be relatively strong on some abundant taxa, including a species of *Spartobacteria*, which was the most common OTU in our samples. Spartobacteria and other members of the Verrucomicrobia are ubiquitous and numerically dominant members of soil communities (Bergmann et al., 2011). Other taxa strongly affected by tillage (i.e., consistently over‐represented in no‐till fields) included a member of the Chitinophagaceae and strains of both Rhizobiales and *Nitrospira*. Nutrient effects may drive differences in the abundance of these taxa across fields, rather than direct disturbance by soil tillage. While not measured, the no‐till fields appeared to have higher crop residue and did have marginally higher levels of organic carbon (Appendix Figure S4). High levels of Chitinophagaceae in soil has been associated with increased activity of β‐glucosidase (Bailey, Fansler, Stegen, & McCue, 2013), an important enzyme associated with the breakdown of cellulose; note, though, that another study (de Vries, Schöler, Ertl, Xu, & Schloter, 2015) did not find differences in cellulose degrading gene copy number in conventionally tilled versus no‐till soils. The higher copy number of genes associated with nitrogen fixation in no‐till soils (Figure 3) may be associated with the high abundance of nitrogen‐fixing *Rhizobiales*. And while no annotation terms were explicitly related to nitrification, the difference in Nitrospira, a known nitrifier (Daims et al., 2015), abundance across tillage type further supports the result that tillage affects the abundance of genes responsible for multiple steps of the nitrogen cycle (Figure 3). Fewer OTUs were consistently biased toward higher abundance in conventionally tilled soil compared to no‐till soil. A strain of Arthrobacter was the bacterium most associated with tilled soil. Arthrobacter is common in soil generally, and some strains are able to metabolize toxins, including agricultural pesticides (Turnbull, Ousley, Walker, Shaw, & Morgan, 2001).

Interestingly, there were clear biases in the number of taxa across several phyla, with some being over or under‐represented in each field type. Taxa within the Actinobacteria, Firmicutes, and Planctomycetes all tended to be more abundant in no‐till fields while the Bacteroidetes and Proteobacteria had the opposite pattern (Figure S1). While it is difficult to make generalizations at the level of bacterial phyla, this result does suggest the potential for broad‐scale differences in phylum‐level adaptation to disturbance and levels of soil nutrients.

We predicted that tillage would cause increased species turnover from year to year. Increased turnover would suggest that communities are less capable of evolving co‐adaptive complexes (Mueller & Sachs, 2015), and this in turn would suggest that tillage may have a long‐term detrimental impact on plant health and yield. However, we found no evidence of a difference in species turnover between fields of different types. While this result does not suggest that soil disturbance has no role in preventing co‐adaptation, it does suggest that disturbance itself may not have the largest impact on community composition. Note, though, that rainfall differences between the 2 years of this study were substantial, and microbial communities are capable of rapid species turnover (Schmidt et al., 2007), and thus, these and other factors may have masked biologically relevant patterns of species turnover linked to soil disturbance. Furthermore, our data only examined DNA, that is, the full complement of what is in the soil regardless of activity (i.e., including diapausing individuals). It is quite possible that an examination of transcribed RNA (whether total or 16S rRNA) would show much more dramatic turnover (Aanderud & Lennon, 2011). Our results show a strong effect of soil tillage on chemical resource levels instead of a direct effect of tillage on community composition. The shotgun sequencing data indicate differences between field types in the number of reads implicated in nitrogen cycling (Figure 2), thus implying a causal relationship between nutrient levels and changes in the functional repertoire of bacterial communities. These results lead to the prediction that microbial community change due to soil disturbance is mediated by the effect of disturbance on nutrient levels, which has already been well established (Gersmehl, 1978; Phillips et al., 1980; Triplett & Dick, 2008). If this prediction is true, then tillage may exacerbate changes in soil nutrients by both increasing nutrient loss due to runoff while also leading to changes in the microbial communities that cycle the nutrients. Increasing our understanding of the relationship between crop health, soil disturbance, soil nutrients, and the soil microbiome is an emerging field that will likely increase in importance as ever‐increasing human population growth puts increasing demands on crop yield.

## Conflicts of Interest

The authors declare no conflict of interest regarding this work or its conclusions.
