# Supplementary material for: Microbial community responses to soil tillage and crop rotation in a corn/soybean agroecosystem
Source: Ecol Evol. 2016 Oct 14;6(22):8075–84. doi: 10.1002/ece3.2553 (PMC5108259; doi:10.1002/ece3.2553)
Supplement: Supplementary file 1 [file ECE3-6-8075-s001.docx]

Appendix:

Order of contents:

- Detailed Shotgun Library Preparation Methods
- Supplementary Tables S1-S9
- Supplementary Figures S1-S4

Detailed Shotgun Library Preparation Methods:

1.1ug of gDNA from each of the forty samples above was resuspended in a final volume of 130ul and sheared on the Covaris E220 instrument in individual microTUBEs using the following settings:

PIP: 105W
Duty Factor: 5%
Cycles per burst: 200
Treatment time: 80 seconds

Fragmented samples were then arrayed into an AB1127 plate and purified using a 0.6X ratio of AmpureXP beads. Samples were eluted in a final volume of 50ul of EB buffer and assayed on the TapeStation using D1K HS tapes.

Next, the fragmented gDNA samples were prepared into DNASeq libraries using the SWHT method on the BioMekFXp using Bioo Scientific NextFlex chemistry and NextFlex-96 DNA barcodes. The large size selection option (350-750bp) was selected. The pre-enriched libraries were eluted in a final volume of 20ul EB buffer. For enrichment, 10ul of pre-enriched library was used as template in a 12 cycle amplification. Following enrichment, the libraries were purified using a 0.8X ratio of AmpureXP beads and eluted in a final volume of 30ul EB buffer. The libraries were then assayed on the TapeStation using D1K HS tapes.

Libraries were then pooled and sequenced in one HiSeq 100bp PE lane.

Table S1. Basic information about the fields used in this study.

| **Field** | **Crop - 2012** | **Crop - 2013** | **Tilled** | **Latitude** | **Longitude** |
| --- | --- | --- | --- | --- | --- |
| 1 | Corn | Soy | No | 39°45'55.38N | 84°50'52.65W |
| 2 | Soy | Corn | No | 39°46'25.63N | 84°48'57.99W |
| 3 | Corn | Soy | No | 39°46'2.51N | 84°50'3.29W |
| 4 | Soy | --- | No | 39°46'25.73N | 84°49'42.96W |
| 5 | Corn | Soy | No | 39°53'58.76N | 85°00'16.77W |
| 6 | Soy | corn | No | 39°53'58.83N | 85°00'28.38W |
| 7 | corn | Soy | No | 39°53'15.8N | 84°58'53.08W |
| 8 | Soy | Corn | No | 39°53'24.96N | 84°58'57.3W |
| 9 | Corn | Soy | No | 39°56'49.01N | 84°48'52.84W |
| 10 | Soy | --- | No | 39°56'48.71N | 84°48'46.61W |
| 11 | Corn | Soy | Yes | 39°55'47.61N | 84°45'51.69W |
| 12 | Soy | Corn | Yes | 39°55'50.72"N | 84°46'3.11"W |
| 13 | Corn | Corn | Yes | 39°48'45.52"N | 84°54'38.58"W |
| 14 | Soy | Corn | Yes | 39°48'4.11N | 84°55'16.86W |
| 15 | Corn | Soy | Yes | 39°27'33.57N | 84°57'7.87W |
| 16 | Soy | Corn | Yes | 39°27'41.28N | 84°56'35.27W |
| 17 | Corn | Soy | Yes | 39°52'45.06N | 84°58'45.30W |
| 18 | Soy | Corn | Yes | 39°52'34.67N | 84°58'43.92W |
| 19 | Corn | Soy | Yes | 39°52'34.07N | 84°58'54.35W |
| 20 | Soy | Corn | Yes | 39°52'46.99N | 84°58'55.84W |

Table S2. Primer barcodes used for each sample. The forward primer in all 16S rRNA sequencing was 515F.

| Year | Field | R Primer | Barcode |
| --- | --- | --- | --- |
| 2012 | 1 | 806r_1 | TCCCTTGTCTCC |
| 2012 | 2 | 806r_2 | ACGAGACTGATT |
| 2012 | 3 | 806r_3 | GCTGTACGGATT |
| 2012 | 4 | 806r_4 | ATCACCAGGTGT |
| 2012 | 5 | 806r_5 | TGGTCAACGATA |
| 2012 | 6 | 806r_6 | ATCGCACAGTAA |
| 2012 | 7 | 806r_7 | GTCGTGTAGCCT |
| 2012 | 8 | 806r_8 | AGCGGAGGTTAG |
| 2012 | 9 | 806r_9 | ATCCTTTGGTTC |
| 2012 | 10 | 806r_10 | TACAGCGCATAC |
| 2012 | 11 | 806r_11 | ACCGGTATGTAC |
| 2012 | 12 | 806r_12 | AATTGTGTCGGA |
| 2012 | 13 | 806r_13 | TGCATACACTGG |
| 2012 | 14 | 806r_14 | AGTCGAACGAGG |
| 2012 | 15 | 806r_15 | ACCAGTGACTCA |
| 2012 | 16 | 806r_16 | GAATACCAAGTC |
| 2012 | 17 | 806r_17 | GTAGATCGTGTA |
| 2012 | 18 | 806r_18 | TAACGTGTGTGC |
| 2012 | 19 | 806r_19 | CATTATGGCGTG |
| 2012 | 20 | 806r_20 | CCAATACGCCTG |
| 2013 | 1 | 806r_21 | GATCTGCGATCC |
| 2013 | 2 | 806r_22 | CAGCTCATCAGC |
| 2013 | 3 | 806r_23 | CAAACAACAGCT |
| 2013 | 5 | 806r_24 | GCAACACCATCC |
| 2013 | 6 | 806r_25 | GCGATATATCGC |
| 2013 | 7 | 806r_26 | CGAGCAATCCTA |
| 2013 | 8 | 806r_27 | AGTCGTGCACAT |
| 2013 | 9 | 806r_28 | GTATCTGCGCGT |
| 2013 | 11 | 806r_29 | CGAGGGAAAGTC |
| 2013 | 12 | 806r_30 | CAAATTCGGGAT |
| 2013 | 13 | 806r_31 | AGATTGACCAAC |
| 2013 | 14 | 806r_32 | AGTTACGAGCTA |
| 2013 | 15 | 806r_33 | GCATATGCACTG |
| 2013 | 16 | 806r_34 | CAACTCCCGTGA |
| 2013 | 17 | 806r_35 | TTGCGTTAGCAG |
| 2013 | 18 | 806r_36 | TACGAGCCCTAA |
| 2013 | 19 | 806r_37 | CACTACGCTAGA |
| 2013 | 20 | 806r_38 | TGCAGTCCTCGA |

Table S3. Sequencing information and accession numbers for all fields sampled. Base-pair (bp) counts and read numbers include only those sequences that passed all quality control filters.

| **Field** | **16S reads 2012** | **16S reads 2013** | **Shotgun bp 2012** | **Shotgun reads 2012** | **Shotgun bp 2013** | **Shotgun reads 2013** | **MgRAST ID 2012** | **MgRAST ID 2013** |
| --- | --- | --- | --- | --- | --- | --- | --- | --- |
| 1 | 282,566 | 361,937 | 2,937,315,025 | 29,058,789 | 3,813,242,064 | 37,717,342 | 4552607.3 | 4552626.3 |
| 2 | 308,158 | 279,806 | 2,647,555,981 | 26,201,119 | 3,644,801,503 | 36,009,146 | 4552609.3 | 4552630.3 |
| 3 | 227,720 | 295,724 | 2,452,638,515 | 24,272,188 | 1,551,039,149 | 15,338,248 | 4552610.3 | 4552631.3 |
| 4 | 250,897 |  | 1,432,525,102 | 14,174,970 |  |  | 4552611.3 |  |
| 5 | 226,838 | 266,702 | 1,186,464,826 | 11,739,217 | 1,533,060,133 | 15,163,928 | 4552612.3 | 4552632.3 |
| 6 | 258,535 | 254,984 | 3,171,919,697 | 31,391,317 | 1,437,615,180 | 14,220,298 | 4552613.3 | 4552633.3 |
| 7 | 252,215 | 218,732 | 1,627,589,348 | 16,105,725 | 1,270,377,420 | 12,563,469 | 4552614.3 | 4552634.3 |
| 8 | 238,603 | 244,908 | 2,230,030,788 | 22,069,730 | 1,350,535,185 | 13,345,371 | 4552615.3 | 4552635.3 |
| 9 | 265,440 | 264,114 | 1,475,556,590 | 14,599,171 | 1,650,655,226 | 16,318,159 | 4552616.3 | 4552636.3 |
| 10 | 260,077 |  | 1,431,745,529 | 14,169,028 |  |  | 4552597.3 |  |
| 11 | 282,682 | 260,534 | 1,235,728,039 | 12,229,474 | 1,144,230,699 | 11,311,337 | 4552598.3 | 4552617.3 |
| 12 | 256,901 | 307,700 | 2,976,499,049 | 29,455,785 | 1,756,407,594 | 17,335,758 | 4552599.3 | 4552618.3 |
| 13 | 249,492 | 274,786 | 2,918,474,743 | 28,877,883 | 1,598,448,932 | 15,798,980 | 4552600.3 | 4552619.3 |
| 14 | 285,757 | 291,275 | 1,596,035,427 | 15,793,481 | 1,787,588,588 | 17,680,870 | 4552601.3 | 4552620.3 |
| 15 | 304,141 | 278,863 | 1,343,608,914 | 13,294,138 | 2,317,770,032 | 22,931,228 | 4552602.3 | 4552621.3 |
| 16 | 242,004 | 251,895 | 1,754,754,798 | 17,361,712 | 1,888,167,542 | 18,668,477 | 4552603.3 | 4552622.3 |
| 17 | 261,746 | 257,631 | 1,036,580,474 | 10,258,094 | 1,891,570,794 | 18,674,403 | 4552604.3 | 4552623.3 |
| 18 | 276,964 | 263,186 | 4,454,201 | 44,065 | 3,617,528,765 | 35,762,397 | 4552605.3 | 4552624.3 |
| 19 | 277,815 | 270,358 | 2,921,679,340 | 28,897,599 | 1,838,277,364 | 18,164,258 | 4552606.3 | 4552625.3 |
| 20 | 238,510 | 260,464 | 1,286,824,690 | 12,735,016 | 3,363,860,585 | 33,236,811 | 4552608.3 | 4552627.3 |

Table S4. Shotgun-Functional/Shotgun-Species/16S rRNA OTU richness variation across year, tillage and crop type. Function richness is the number of distinct ontology categories annotated from the shotgun sequencing. Shotgun richness is the number of taxa annotated from the shotgun sequencing. OTU richness is the number of operational taxonomic units at the 97% identity cutoff using 16S rRNA.

|  |  |  | **Function Richness** | | | **Shotgun Richness** | | | **OTU Richness** | | |
| --- | --- | --- | --- | --- | --- | --- | --- | --- | --- | --- | --- |
| **Year** | **Tilled** | **Crop** | **Mean** | **St. Dev.** | **N** | **Mean** | **St. Dev.** | **N** | **Mean** | **St. Dev.** | **N** |
| 2012 | No | Corn | 1,073 | 3 | 5 | 1,334 | 36 | 5 | 22,835 | 1,473 | 5 |
| 2012 | No | Soy | 1,072 | 5 | 5 | 1,309 | 45 | 5 | 23,656 | 1,384 | 5 |
| 2012 | Yes | Corn | 1,080 | 2 | 5 | 1,356 | 50 | 5 | 22,914 | 1,823 | 5 |
| 2012 | Yes | Soy | 1,078 | 6 | 4 | 1,368 | 11 | 4 | 23,431 | 2,613 | 5 |
| 2013 | No | Corn | 1,076 | 6 | 3 | 1,200 | 16 | 3 | 23,604 | 1,247 | 3 |
| 2013 | No | Soy | 1,070 | 6 | 5 | 1,244 | 39 | 5 | 22,747 | 1,417 | 5 |
| 2013 | Yes | Corn | 1,079 | 4 | 6 | 1,240 | 52 | 6 | 22,278 | 1,550 | 6 |
| 2013 | Yes | Soy | 1,078 | 4 | 4 | 1,285 | 60 | 4 | 24,350 | 1,021 | 4 |
|  |  | **Avg** | **1,076** |  |  | **1,292** |  |  | **23,227** |  |  |

|  |  |  | **OTU Richness (no singletons)** | | |
| --- | --- | --- | --- | --- | --- |
| **Year** | **Tilled** | **Crop** | **Mean** | **St. Dev.** | **N** |
| 2012 | No | Corn | 14,445 | 621 | 5 |
| 2012 | No | Soy | 15,275 | 583 | 5 |
| 2012 | Yes | Corn | 15,543 | 905 | 5 |
| 2012 | Yes | Soy | 15,302 | 1,404 | 5 |
| 2013 | No | Corn | 14,260 | 1,127 | 3 |
| 2013 | No | Soy | 13,674 | 948 | 5 |
| 2013 | Yes | Corn | 13,859 | 1,044 | 6 |
| 2013 | Yes | Soy | 14,447 | 521 | 4 |
|  |  | **Avg** | 14,601 |  |  |

Table S5. Inverse Simpson’s index variation across year, tillage and crop type. Function diversity is the number of distinct ontology categories annotated from the shotgun sequencing. Shotgun diversity is the number of taxa annotated from the shotgun sequencing. OTU diversity is the number of operational taxonomic units at the 97% identity cutoff using 16S rRNA.

|  |  |  | **Function Diversity** | | | **Shotgun Diversity** | | | **OTU Diversity** | | |
| --- | --- | --- | --- | --- | --- | --- | --- | --- | --- | --- | --- |
| **Year** | **Tilled** | **Crop** | **Mean** | **St. Dev.** | **N** | **Mean** | **St. Dev.** | **N** | **Mean** | **St. Dev.** | **N** |
| 2012 | No | Corn | 282 | 1 | 5 | 60 | 8 | 5 | 303 | 99 | 5 |
| 2012 | No | Soy | 283 | 2 | 5 | 51 | 10 | 5 | 408 | 76 | 5 |
| 2012 | Yes | Corn | 283 | 1 | 5 | 73 | 16 | 5 | 399 | 53 | 5 |
| 2012 | Yes | Soy | 284 | 2 | 4 | 71 | 6 | 4 | 471 | 42 | 5 |
| 2013 | No | Corn | 283 | 1 | 3 | 46 | 3 | 3 | 446 | 67 | 3 |
| 2013 | No | Soy | 284 | 2 | 5 | 48 | 3 | 5 | 370 | 80 | 5 |
| 2013 | Yes | Corn | 285 | 1 | 6 | 53 | 10 | 6 | 465 | 73 | 6 |
| 2013 | Yes | Soy | 284 | 1 | 4 | 53 | 12 | 4 | 504 | 68 | 4 |
|  |  | **Avg** | **284** |  |  | **57** |  |  | **421** |  |  |

|  |  |  | **OTU Diversity (no singletons)** | | |
| --- | --- | --- | --- | --- | --- |
| **Year** | **Tilled** | **Crop** | **Mean** | **St. Dev.** | **N** |
| 2012 | No | Corn | 279 | 89 | 5 |
| 2012 | No | Soy | 375 | 71 | 5 |
| 2012 | Yes | Corn | 369 | 45 | 5 |
| 2012 | Yes | Soy | 434 | 38 | 5 |
| 2013 | No | Corn | 408 | 61 | 3 |
| 2013 | No | Soy | 341 | 73 | 5 |
| 2013 | Yes | Corn | 430 | 66 | 6 |
| 2013 | Yes | Soy | 459 | 60 | 4 |
|  |  | **Avg** | 387 |  |  |

Table S6. 16S rRNA copy number variation across year, tillage, and crop type.

|  |  |  | **Copies/mg dry soil** | | |
| --- | --- | --- | --- | --- | --- |
|  |  |  | Mean | St. Dev. | N |
| 2012 | No | Corn | 32,809,485 | 13,107,893 | 5 |
| 2012 | No | Soy | 45,457,543 | 6,347,748 | 5 |
| 2012 | Yes | Corn | 32,971,059 | 17,644,248 | 5 |
| 2012 | Yes | Soy | 35,082,344 | 11,404,740 | 5 |
| 2013 | No | Corn | 21,268,623 | 5,527,885 | 3 |
| 2013 | No | Soy | 16,881,864 | 13,100,101 | 5 |
| 2013 | Yes | Corn | 15,789,865 | 8,447,298 | 6 |
| 2013 | Yes | Soy | 17,713,326 | 5,255,216 | 4 |
|  |  | **Avg** | 27,246,764 |  |  |

Table S7. Repeated-measures ANOVA test statistic information for the three measures of diversity used, shotgun-function/shotgun-species/16S rRNA OTUs, and the two measures of alpha diversity, richness and inverse Simpson’s diversity measure.

|  |  | Richness | | | Diversity | | |
| --- | --- | --- | --- | --- | --- | --- | --- |
|  |  | F | df | P | F | df | P |
| Function | Till | 1.34 | 1,31 | 0.26 | 1.52 | 1,31 | 0.23 |
|  | Crop | 3.03 | 1,31 | 0.09 | 2.85 | 1,31 | 0.1 |
|  | Till x Crop | 2.12 | 1,31 | 0.16 | 2.12 | 1,31 | 0.16 |
|  |  |  |  |  |  |  |  |
| Species | Till | 0 | 1,31 | 0.99 | 0.528 | 1,31 | 0.47 |
|  | Crop | 0.28 | 1,31 | 0.6 | 0.769 | 1,31 | 0.39 |
|  | Till x Crop | 0.85 | 1,31 | 0.36 | 0.572 | 1,31 | 0.46 |
|  |  |  |  |  |  |  |  |
| OTU | Till | 8.48 | 1,32 | 0.007 | 0.26 | 1,32 | 0.64 |
|  | Crop | 1.6 | 1,32 | 0.22 | 2.33 | 1,32 | 0.14 |
|  | Till x Crop | 1.2 | 1,32 | 0.28 | 0.08 | 1,32 | 0.78 |

Table S8. Permutational ANOVA results. These results correspond to the community data graphed in Figure 1.

|  |  | F | df | P | R^2^ |
| --- | --- | --- | --- | --- | --- |
| 2012 OTU | Till | 4.1 | 1,19 | 0.002 | 0.18 |
|  | Crop | 1.6 | 1,19 | 0.08 | 0.07 |
|  | Till x Crop | 1.5 | 1,19 | 0.11 | 0.06 |
|  |  |  |  |  |  |
| 2013 OTU | Till | 1.81 | 1,17 | 0.02 | 0.1 |
|  | Crop | 0.99 | 1,17 | 0.42 | 0.06 |
|  | Till x Crop | 0.87 | 1,17 | 0.63 | 0.05 |
|  |  |  |  |  |  |
| Function 2012 | Till | 6.86 | 1,19 | 0.001 | 0.26 |
|  | Crop | 4.02 | 1,19 | 0.002 | 0.15 |
|  | Till x Crop | 0.93 | 1,19 | 0.44 | 0.03 |
|  |  |  |  |  |  |
| Function 2013 | Till | 2.11 | 1,17 | 0.04 | 0.12 |
|  | Crop | 1.02 | 1,17 | 0.38 | 0.06 |
|  | Till x Crop | 0.54 | 1,17 | 0.87 | 0.03 |
|  |  |  |  |  |  |
| OTU 2012 | Till | 4.46 | 1,19 | 0.003 | 0.19 |
| (no singletons) | Crop | 1.66 | 1,19 | 0.07 | 0.07 |
|  | Till x Crop | 1.53 | 1,19 | 0.1 | 0.06 |
|  |  |  |  |  |  |
| OTU 2013 | Till | 1.88 | 1,17 | 0.02 | 0.11 |
| (no singletons) | Crop | 0.98 | 1,17 | 0.42 | 0.06 |
|  | Till x Crop | 0.85 | 1,17 | 0.65 | 0.05 |

Table S9.

| **Simper TopTen** | **No-Till - Till*** | **Taxonomy** |
| --- | --- | --- |
| 1 | 3392.9875 | Bacteria(100);Acidobacteria(100);Acidobacteria_Gp6(100);Acidobacteria_Gp6_order_incertae_sedis(100);Acidobacteria_Gp6_family_incertae_sedis(100);Gp6(100); |
| 2 | 967 | Bacteria(100);Verrucomicrobia(100);Spartobacteria(100);Spartobacteria_order_incertae_sedis(100);Spartobacteria_family_incertae_sedis(100);Spartobacteria_genera_incertae_sedis(100); |
| 3 | 1751.4 | Bacteria(100);unclassified(100);unclassified(100);unclassified(100);unclassified(100);unclassified(100); |
| 4 | -770.2375 | Bacteria(100);Acidobacteria(100);Acidobacteria_Gp1(100);Acidobacteria_Gp1_order_incertae_sedis(100);Acidobacteria_Gp1_family_incertae_sedis(100);Gp1(100); |
| 5 | 265.825 | Bacteria(100);Acidobacteria(100);Acidobacteria_Gp4(100);Acidobacteria_Gp4_order_incertae_sedis(100);Acidobacteria_Gp4_family_incertae_sedis(100);Gp4(100); |
| 6 | 1129.6125 | Bacteria(100);Verrucomicrobia(100);Subdivision3(100);Subdivision3_order_incertae_sedis(100);Subdivision3_family_incertae_sedis(100);3_genus_incertae_sedis(100); |
| 7 | -287.2125 | Bacteria(100);Bacteroidetes(100);Sphingobacteria(100);Sphingobacteriales(100);Chitinophagaceae(100);unclassified(100); |
| 8 | -11.7375 | Bacteria(100);Bacteroidetes(100);unclassified(100);unclassified(100);unclassified(100);unclassified(100); |
| 9 | 336.8625 | Bacteria(100);Proteobacteria(100);Gammaproteobacteria(100);unclassified(100);unclassified(100);unclassified(100); |
| 10 | 94.725 | Bacteria(100);Proteobacteria(100);Alphaproteobacteria(100);Rhizobiales(100);unclassified(100);unclassified(100); |
| **Indicators of No-Till** | **No-Till - Till*** | **Taxonomy** |
| 1 | 0.7125 | Bacteria(100);Acidobacteria(100);Acidobacteria_Gp19(100);Acidobacteria_Gp19_order_incertae_sedis(100);Acidobacteria_Gp19_family_incertae_sedis(100);Gp19(100); |
| 2 | 1.6 | Bacteria(100);Acidobacteria(100);Acidobacteria_Gp21(100);Acidobacteria_Gp21_order_incertae_sedis(100);Acidobacteria_Gp21_family_incertae_sedis(100);Gp21(100); |
| 3 | 0.5375 | Bacteria(100);Verrucomicrobia(100);Subdivision5(100);Subdivision5_order_incertae_sedis(100);Subdivision5_family_incertae_sedis(100);5_genus_incertae_sedis(100); |
| 4 | 1.0125 | Bacteria(100);Chlamydiae(100);Chlamydiae(100);Chlamydiales(100);Simkaniaceae(100);Simkania(100); |
| 5 | 0.9875 | Bacteria(100);Chlorobi(100);Ignavibacteria(100);Ignavibacteriales(100);Ignavibacteriaceae(100);Ignavibacterium(100); |
| 6 | 0.3375 | Bacteria(100);Firmicutes(100);Clostridia(100);Clostridiales(100);Peptostreptococcaceae(100);Tepidibacter(100); |
| **Indicators of Till** | **No-Till - Till*** | **Taxonomy** |
| 1 | -1 | Bacteria(100);Actinobacteria(100);Actinobacteria(100);Actinomycetales(100);Micrococcaceae(100);Renibacterium(100); |
| 2 | -12.5 | Bacteria(100);Actinobacteria(100);Actinobacteria(100);Actinomycetales(100);Pseudonocardiaceae(100);Lechevalieria(100); |
| 3 | -5.125 | Bacteria(100);Proteobacteria(100);Alphaproteobacteria(100);Sphingomonadales(100);Sphingomonadaceae(100);Sphingopyxis(100); |
| 4 | -1.85 | Bacteria(100);Proteobacteria(100);Alphaproteobacteria(100);Rhizobiales(100);Aurantimonadaceae(100);Aurantimonas(100); |
| 5 | -2.8625 | Bacteria(100);Proteobacteria(100);Gammaproteobacteria(100);Xanthomonadales(100);Xanthomonadaceae(100);Luteibacter(100); |
| 6 | -1.2 | Bacteria(100);Proteobacteria(100);Alphaproteobacteria(100);Rhodospirillales(100);Rhodospirillaceae(100);Azospirillum(100); |
| 7 | -1.025 | Bacteria(100);Firmicutes(100);Bacilli(100);Bacillales(100);Paenibacillaceae_2(100);Oxalophagus(100); |
| 8 | -0.775 | Bacteria(100);Actinobacteria(100);Actinobacteria(100);Actinomycetales(100);Micromonosporaceae(100);Allocatelliglobosispora(100); |
| 9 | -1.9875 | Bacteria(100);Proteobacteria(100);Betaproteobacteria(100);Methylophilales(100);Methylophilaceae(100);Methylobacillus(100); |
| 10 | -1.1125 | Bacteria(100);Actinobacteria(100);Actinobacteria(100);Actinomycetales(100);Microbacteriaceae(100);Frondihabitans(100); |
| 11 | -1.7375 | Bacteria(100);Proteobacteria(100);Betaproteobacteria(100);Burkholderiales(100);Comamonadaceae(100);Pseudorhodoferax(100); |
| 12 | -1.5625 | Bacteria(100);Actinobacteria(100);Actinobacteria(100);Actinomycetales(100);Micromonosporaceae(100);Verrucosispora(100); |
| 13 | -0.7875 | Bacteria(100);Proteobacteria(100);Alphaproteobacteria(100);Rhizobiales(100);Beijerinckiaceae(100);Methylocella(100); |
| 14 | -0.6625 | Bacteria(100);Firmicutes(100);Negativicutes(100);Selenomonadales(100);Veillonellaceae(100);Veillonellaceae_genus_incertae_sedis(100); |
| 15 | -0.4625 | Bacteria(100);Proteobacteria(100);Gammaproteobacteria(100);Pseudomonadales(100);Pseudomonadaceae(100);Rhizobacter(100); |
| 16 | -0.3375 | Bacteria(100);Actinobacteria(100);Actinobacteria(100);Actinomycetales(100);Micromonosporaceae(100);Couchioplanes(100); |
| 17 | -1.0375 | Bacteria(100);Proteobacteria(100);Alphaproteobacteria(100);Rhizobiales(100);Brucellaceae(100);unclassified(100); |
| 18 | -0.85 | Bacteria(100);Proteobacteria(100);Betaproteobacteria(100);Burkholderiales(100);Alcaligenaceae(100);Achromobacter(100); |
| 19 | -0.25 | Bacteria(100);Proteobacteria(100);Betaproteobacteria(100);Burkholderiales(100);Oxalobacteraceae(100);Collimonas(100); |

* The total number of 16S rRNA reads for both 2012 and 2013 in no-till fields minus the total number in conventional till fields.

Figure S1.


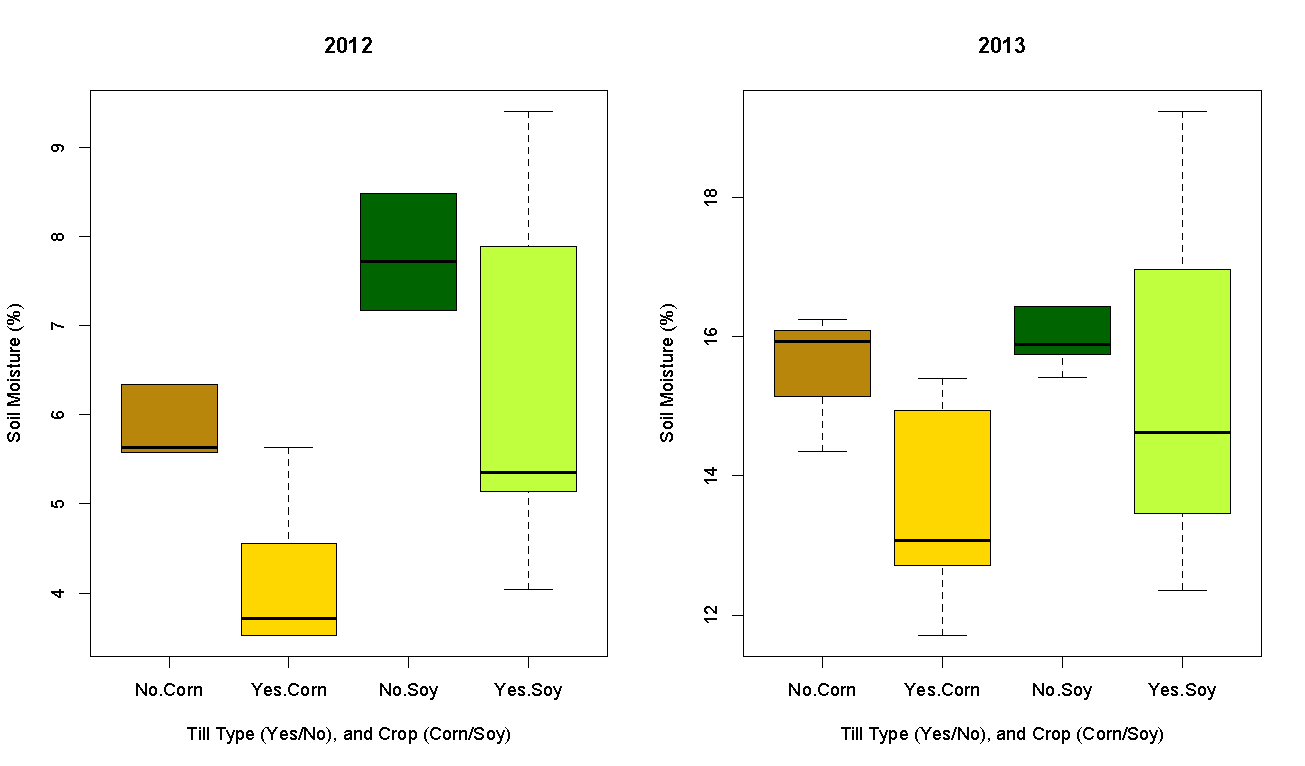


Figure S1. Boxplots of yearly soil moisture (% wet mass that was water) for no-till and conventionally tilled fields planted in either corn or soy in each 2012 (left panel) and 2013 (right panel). Crop had a stronger effect on moisture in each year (F1,16 = 4.9, P = 0.042 for 2012, F1,14 = 4.3, P = 0.056 for 2013) than did tillage type (F1,16 = 4.2, P = 0.058 for 2012, F1,14 = 3.5, P = 0.084 for 2013); there was no interaction in either year.

Figure S2.


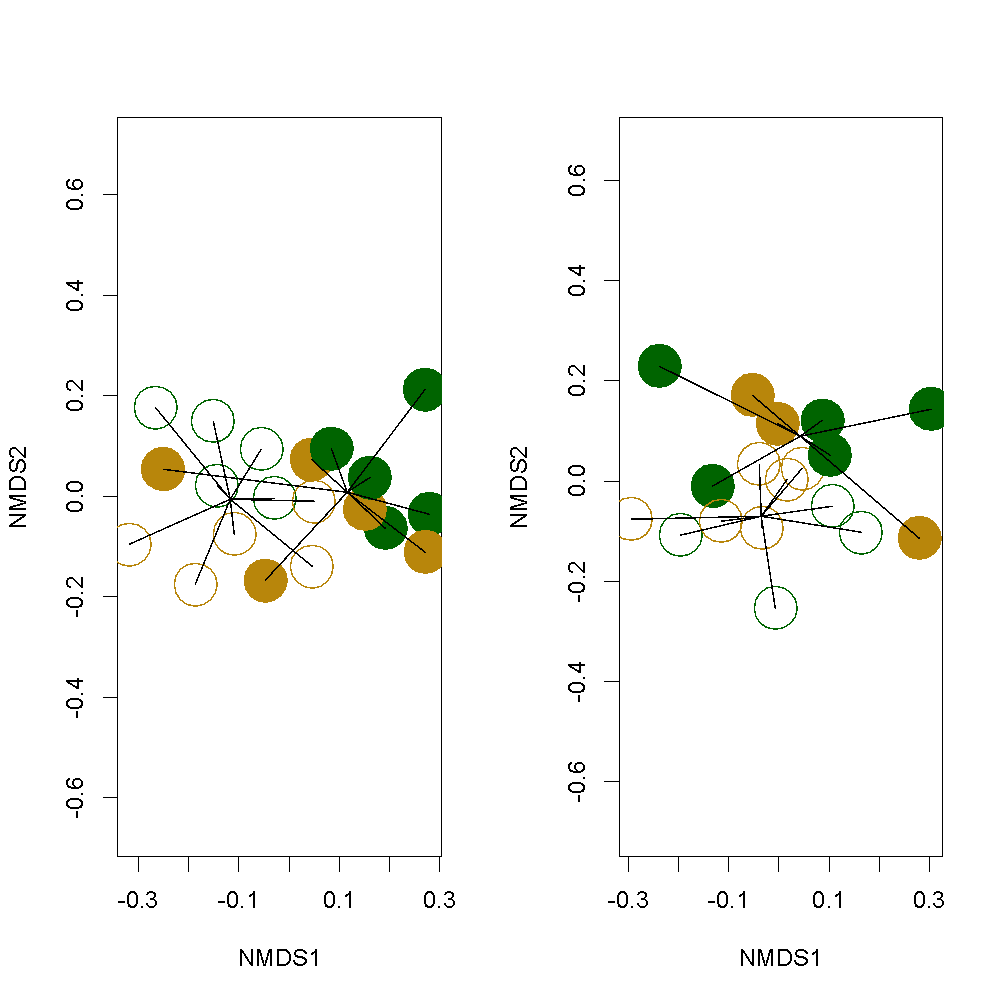


Figure S2. Ordination (NMDS) plots using OTU data from 2012 (left) and 2013 (right) in which singleton sequences were excluded – filled symbols represent no-till fields, open symbols conventionally tilled fields, yellow symbols are corn and green are soybean. The effect of tillage was significant in both years in a permutational ANOVA, but crop and the interaction between crop and tillage type were not significant. Full analysis results are available in Table S8.

Figure S3.

Figure S3. The frequency of biased taxa in no-till and conventionally tilled fields, within each of the phyla listed. Significance values are listed for phyla where more/fewer than expected taxa showed a bias in no-till fields compared to the expectation of equal frequencies for no-till and conventionally tilled fields.

Figure S4.

Figure S4. Boxplots of six soil nutrient in each treatment (across years). Boxes with dark fill represent no -fields, while light fill is for conventional till fields. Yellow fill is for corn fields, while green is for soy fields. While no nutrient was significantly different with regard to either till type or crop type in repeated measures ANOVA (see main text), most nutrients have a higher median and mean in no-till fields, regardless of crop.
